# Protein phosphatase 2A anchoring disruptor gene therapy for familial dilated cardiomyopathy

**DOI:** 10.1016/j.omtm.2024.101233

**Published:** 2024-03-11

**Authors:** Xueyi Li, Jinliang Li, Anne-Maj Samuelsson, Hrishikesh Thakur, Michael S. Kapiloff

**Affiliations:** 1 Stanford Cardiovascular Institute, Departments of Ophthalmology and Medicine, Stanford University, Palo Alto, CA 94304, USA

**Keywords:** dilated cardiomyopathy, PP2A, adeno-associated virus, gene therapy

## Abstract

Familial dilated cardiomyopathy is a prevalent cause of heart failure that results from the mutation of genes encoding proteins of diverse function. Despite modern therapy, dilated cardiomyopathy typically has a poor outcome and is the leading cause of cardiac transplantation. The phosphatase PP2A at cardiomyocyte perinuclear mAKAPβ signalosomes promotes pathological eccentric cardiac remodeling, as is characteristic of dilated cardiomyopathy. Displacement of PP2A from mAKAPβ, inhibiting PP2A function in that intracellular compartment, can be achieved by expression of a mAKAPβ-derived PP2A binding domain-derived peptide. To test whether PP2A anchoring disruption would be effective at preventing dilated cardiomyopathy-associated cardiac dysfunction, the adeno-associated virus gene therapy vector AAV9sc.PBD was devised to express the disrupting peptide in cardiomyocytes *in vivo*. Proof-of-concept is now provided that AAV9sc.PBD improves the cardiac structure and function of a cardiomyopathy mouse model involving transgenic expression of a mutant α-tropomyosin E54K *Tpm1* allele, while AAV9sc.PBD has no effect on normal non-transgenic mice. At the cellular level, AAV9sc.PBD restores cardiomyocyte morphology and gene expression in the mutant *Tpm1* mouse. As the mechanism of AAV9sc.PBD action suggests potential efficacy in dilated cardiomyopathy regardless of the underlying etiology, these data support the further testing of AAV9sc.PBD as a broad-based treatment for dilated cardiomyopathy.

## Graphical abstract

**Figure:**
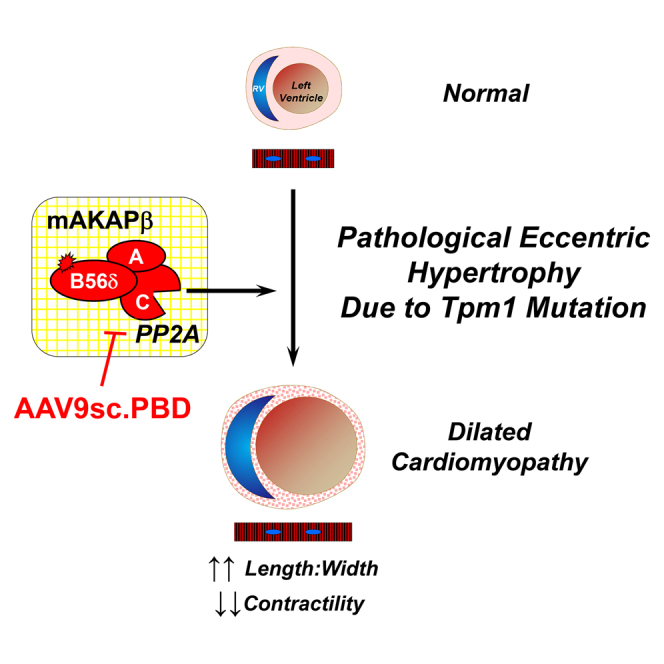

## Teaser

Dr. Kapiloff and his colleagues describe a new gene therapy for familial dilated cardiomyopathy that is etiology agnostic. Expression of a PP2A anchoring disruptor peptide using a cardiomyocyte-directed AAV vector improves the structure and function of the heart in a mouse cardiomyopathy model, providing proof-of-concept for the new therapy.

## Introduction

Non-ischemic dilated cardiomyopathy (DCM) is defined by left ventricular dilatation and reduced contractile function (ejection fraction <45%) in the absence of coronary artery disease.^1^ Current treatment options for DCM include conventional medical and device therapy for heart failure with reduced ejection fraction (HFrEF) and fatal arrhythmia. However, despite modern therapy, a DCM diagnosis incurs a 3-year mortality of 12%–20%.^2^ Importantly, with a prevalence as high as 1 in 250 in the adult population,^3^ DCM comprises a significant cause of cardiovascular mortality and is the most common indication for heart transplantation.^4^

The etiology of DCM is highly heterogeneous. About 30%–50% of DCM cases are genetic, and, notably, mutations in >100 genes have been associated with familial DCM, including those for the sarcomere and costamere, the nuclear lamina and gene expression, calcium handling, extracellular matrix, RNA splicing, and metabolism.^1^ Many therapies under development for familial DCM are directed at restoring the function of mutant genes.^4^ However, due to the diversity and low prevalence of individual DCM mutations, it is challenging to develop a gene replacement or correction therapy that would be broadly applicable across the DCM patient population. Instead, it may be possible to develop broadly indicated treatments targeting the DCM defining phenotype of left ventricular systolic dysfunction and dilatation.

Regardless of the cause, DCM presents as left ventricular dilatation and eccentric cardiac hypertrophy with decreased cardiomyocyte contractile function.^1^^,^^5^ In DCM, ventricular myocytes are both increased in volume and elongated,^6^ a form of cellular hypertrophy featuring the preferential addition of sarcomeres in series and an increase in myocyte length greater than in width. At the organ level, myocyte elongation, in conjunction with myocyte side-to-side slippage,^6^ decreases relative wall thickness, increasing wall stress. Increased wall stress compounds adverse remodeling, worsening underlying defects in myocyte metabolism and contractility, promoting myocyte death and interstitial fibrosis, and contributing to the development of HFrEF. We hypothesize that a gene therapy improving cardiomyocyte morphology, in conjunction with inhibition of other common features of pathological cardiac remodeling, will improve the status of patients with DCM.

**Figure 1:**
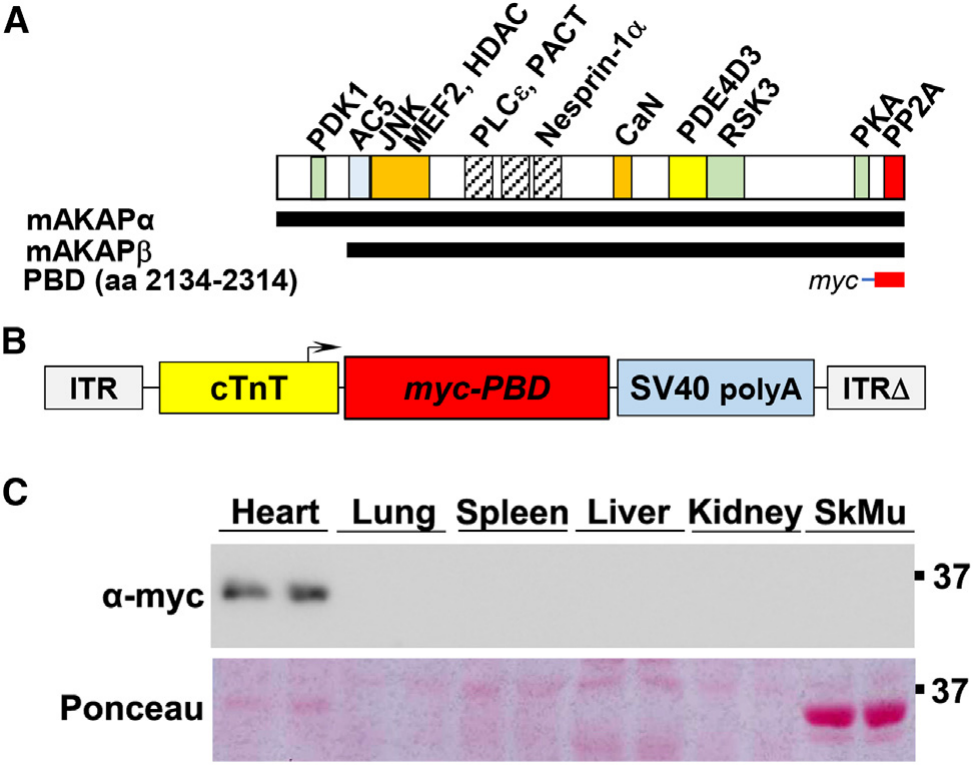
AAV9sc.PBD – a cardiomyocyte-specific gene therapy vector targeting mAKAPβ-PP2A signalosomes (A) mAKAPβ is the alternatively spliced isoform of mAKAP (AKAP6) expressed in striated myocytes and is identical to mAKAPα aa 245–2314 (numbering according to rat protein). Binding sites for direct binding partners that are well-defined are shown; hatched bars are spectrin-like repeats. Nesprin-1α is the integral membrane protein that binds the large mAKAPβ signalosome to the cardiomyocyte nuclear envelope.^10^ Binding partners for mAKAP include nuclear factor of activated T cells (NFAT), serum response factor (SRF), myocyte enhancer factor 2 (MEF2), and hypoxia inducible factor 1α (HIF-1α) transcription factors, class IIa histone deacetylases (HDAC4/5), type 5 adenylyl cyclase (AC5), protein kinase A (PKA), the guanine nucleotide exchange factor Epac1, and phosphodiesterase 4D3 (PDE4D3), which comprise a local cAMP signaling compartment, phospholipase Cε (PLCε), protein kinase Cε (PKCε), protein kinase D (PKD), 3-phosphoinositide-dependent kinase-1 (PDK1), mitogen-activated protein kinase kinase 5 (MEK5), and extracellular-signal-regulated kinase 5 (ERK5), p90 ribosomal S6 kinase type 3 (RSK3), JUN N-terminal kinase (JNK), ras-related protein 1 (Rap1), Seven In Absentia Homolog 1 (SIAH1), prolyl hydroxylases (PHD2/3), von Hippel-Lindau protein (pVHL), phospholamban (PLB), myopodin, calcineurin (CaN), perinuclear ryanodine receptor (RyR2) calcium release channels, and the PACT domain scaffold proteins pericentrin (Pcnt) and A-kinase anchoring protein 9 (AKAP9).^8^^,^^9^ Of the multiple potential local substrates, mAKAPβ-bound PP2A has been shown to dephosphorylate SRF and PDE4D3.^7^^,^^11^ (B) AAV9sc.PBD is a self-complementary serotype 9 gene therapy vector that expresses a myc-tagged PBD peptide under the control of the cardiomyocyte-specific chicken troponin T promoter (cTnT).^7^ (C) An anti-myc tag antibody was used to detect PBD expression by western blot in mice 16 weeks after neonatal AAV9sc.PBD administration. Ponceau, total protein stain showing equal protein loading. SkMu, skeletal muscle. n = 2.

Recently, we described the role in increasing cardiomyocyte length:width ratio of PP2A bound to the perinuclear scaffold protein mAKAPβ (AKAP6β).^7^ At the outer nuclear membrane, mAKAPβ organizes a large multimolecular signaling complex or “signalosome” that integrates upstream signals regulating cardiomyocyte stress-dependent gene expression.^8^ Consistent with the function of mAKAPβ signalosomes in pathological cardiac hypertrophy, loss of cardiomyocyte mAKAPβ expression in mice, both by conditional gene knockout and adeno-associated virus (AAV)-mediated short hairpin RNA (shRNA) expression, inhibited the development of systolic dysfunction and HFrEF following myocardial infarction.^9^ PP2A binds a C-terminal domain of mAKAPβ comprising amino acid residues 2134–2314 (PP2A binding domain, abbreviated PBD, Figure 1A), and PP2A can be displaced from mAKAPβ signalosomes by expression of recombinant PBD peptide.^7^ Like mAKAPβ gene targeting, AAV-mediated PBD expression inhibited the development of systolic dysfunction after myocardial infarction in mice.^7^

The pathophysiologic mechanisms resulting in HFrEF following myocardial infarction differ from those in non-ischemic DCM. However, the role of mAKAPβ-bound PP2A in promoting pathological eccentric hypertrophy suggests that targeting of mAKAPβ-PP2A perinuclear signalosomes may be also beneficial in DCM disease. We now show that expression of the PBD PP2A anchoring disruptor peptide using an AAV gene therapy vector improves the cardiac structure and function of a DCM mouse model, including significantly increased left ventricular systolic function and normalized cardiomyocyte length:width ratio. This study provides proof-of-concept for an approach to DCM therapy based upon targeting mAKAPβ-PP2A signalosomes that is expected to be etiologic independent and may be applicable regardless of DCM mutation.

## Results

### AAV9sc.PBD gene therapy improves the cardiac function of the TM54 DCM mouse

Mutations in the gene *TPM1* encoding the sarcomeric protein α-tropomyosin account for ∼1%–2% of DCM cases.^1^ The well-characterized *Tpm1* E54K DCM mouse model [FVB/N-Tg(Myh6-Tpm1∗E54K), abbreviated “TM54”] is a transgenic in which the indicated α-tropomyosin missense mutant is expressed under the control of the cardiac myocyte-specific α-myosin heavy chain promoter, resulting in replacement of wild-type myocardial α-tropomyosin with E54K mutant protein.^12^ TM54 mice exhibit early onset of a DCM phenotype and heart failure. To test the potential benefit of PBD gene therapy in DCM, TM54 mice were treated at 2–3 days of age with a serotype 9 self-complementary AAV that expresses the mAKAPβ-derived PBD peptide under the control of the cardiac troponin T cardiomyocyte-specific promoter (AAVsc.PBD), resulting in cardiac-specific PBD expression (Figures 1B and 1C). AAV9sc.PBD-treated mice were compared with those administered control AAV9sc.GFP that expresses green fluorescent protein (GFP).

**Figure 2:**
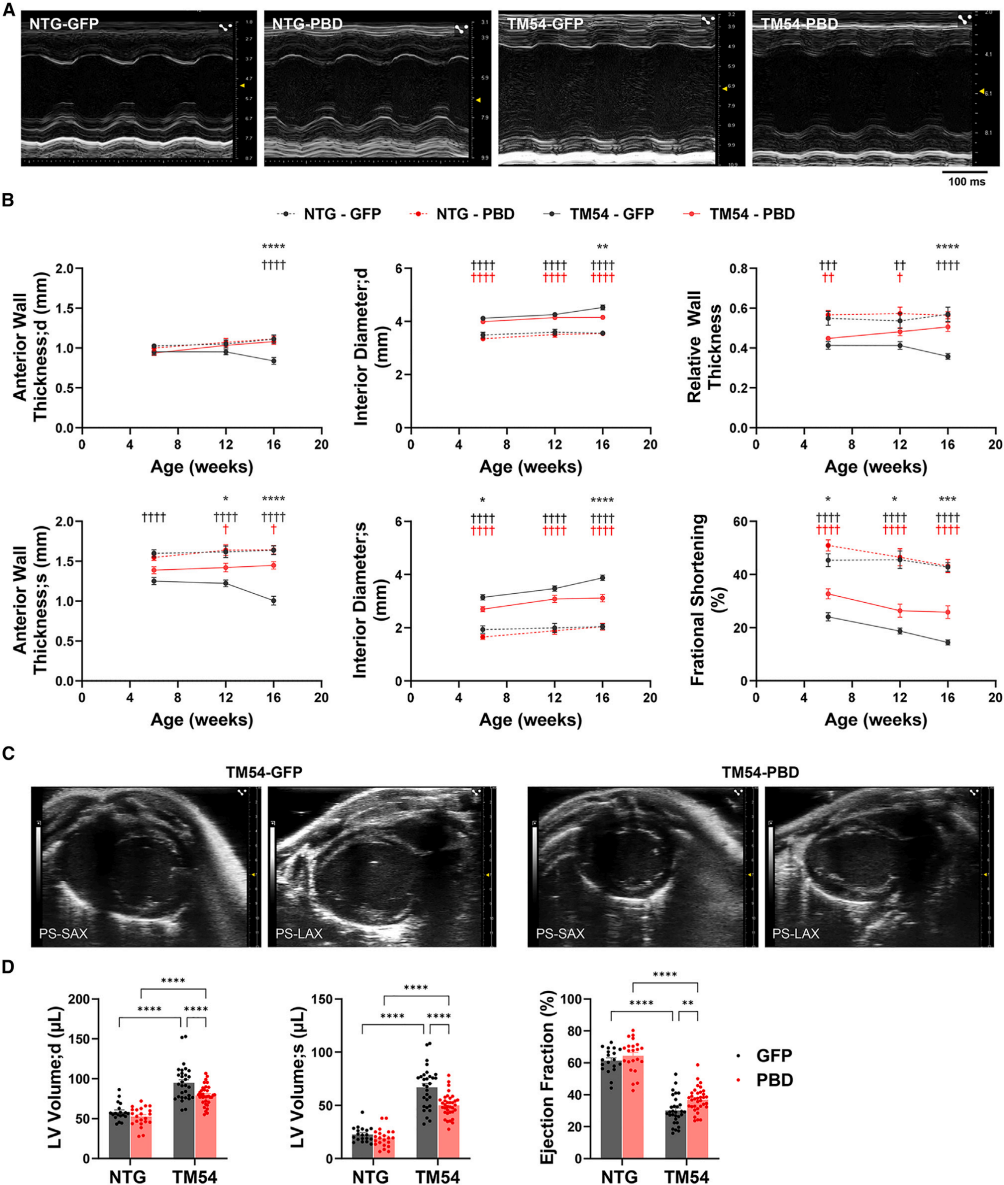
AAV9sc.PBD treatment attenuates eccentric remodeling and systolic dysfunction in TM54 mice Two- to 3-day-old TM54 and non-transgenic (NTG) littermate mice were injected intraperitoneally with 10^11^ vg AAV9sc.PBD (PBD, red data points) or AAV9sc.GFP control (GFP, black data points) (A) Representative M-mode echocardiographic images for 16-week-old mice. (B) Serial M-mode left ventricular echocardiography when mice were 6, 12, and 16 weeks of age. Diameter - d – diastole; systole. ^†^ (in black) TM54-GFP vs. NTG-GFP; ^†^ (in red) TM54-PBD vs. NTG-PBD; ∗ TM54-PBD vs. TM54-GFP; n = NTG-GFP – 13, NTG – PBD – 15; TM54 – GFP – 19; TM54-PBD – 22. (C) Representative B-mode echocardiographic images for 16-week-old mice. (D) Left ventricular volumes and ejection fraction at endpoint based on B-mode images. ∗∗p < 0.01; ∗∗∗∗p < 0.0001; n = NTG-GFP – 19, NTG – PBD – 22; TM54 – GFP – 30; TM54-PBD – 35. Bars are mean ± SEM. Data analyzed by two-way ANOVA and Tukey’s (B) or uncorrected Fisher’s least significant difference (D) post hoc testing.

An initial longitudinal survey by echocardiography of the left ventricle showed that in comparison with non-transgenic (NTG) littermates, TM54 mice administered the control AAV vector exhibited progressively decreased anterior wall thickness, increased chamber diameter, decreased relative wall thickness, and decreased fractional shortening, indicative of the DCM phenotype of ventricular dilatation and systolic dysfunction (Figures 2A and 2B). In contrast, with time, AAV9sc.PBD-treated mice had significantly preserved anterior wall thickness (in both diastole and systole), reduced ventricular dilatation, normalized relative wall thickness, and increased fractional shortening, indicative of an improved cardiac phenotype. As significant differences were evident at the 12- and 16-week time points, 16 weeks was chosen as the endpoint for this study. These and other mice included in this project were comprehensively imaged at endpoint. As in the initial survey, left ventricular structure and function were improved by AAV9sc.PBD treatment as observed by M-mode echocardiography (Table 1). In addition, by B-mode echocardiography, which provides a more complete visualization of the left ventricle, AAV9sc.PBD treatment of the TM54 mice resulted in reduced ventricular volumes in both diastole and systole and a significantly increased left ventricular ejection fraction at endpoint (Figures 2C and 2D; Table 1). Together, these results show that AAV9sc.PBD gene therapy provided a significant preservation of cardiac structure and function, despite the ongoing presence of the mutant sarcomeric protein. Notably, AAV9sc.PBD had no apparent effect on the hearts of NTG normal littermates.

**Table 1:** AAV9sc.PBD treatment improves the cardiac function of the TM54 DCM mouse - echocardiography of 16-week-old mice

	NTG-GFP	NTG-PBD	TM54-GFP	TM54-PBD
n	19	22	30	35
Males (Fraction total)	0.47	0.50	0.40	0.46
Body Weight g	28.6 ± 1.1	27.8 ± 1.0	27.9 ± 0.8	28.0 ± 0.8
Heart Rate BPM	485 ± 8	480 ± 6	475 ± 3	482 ± 5

**M-mode - Left Ventricle**				

Anterior Wall; d mm	1.14 ± 0.05	1.10 ± 0.03	0.93 ± 0.04^†††^	1.10 ± 0.03∗∗∗
Anterior Wall; s mm	1.66 ± 0.04	1.61 ± 0.05	1.13 ± 0.05^†††^	1.40 ± 0.04^††,^∗∗∗
Posterior Wall; d mm	0.84 ± 0.05	0.82 ± 0.03	0.77 ± 0.02	0.95 ± 0.04^††,^∗∗∗
Posterior Wall; s mm	1.32 ± 0.06	1.29 ± 0.05	0.89 ± 0.03^†††^	1.20 ± 0.04∗∗∗
Interior Diameter; d mm	3.64 ± 0.06	3.57 ± 0.06	4.49 ± 0.07^†††^	4.22 ± 0.04^†††,^∗∗
Interior Diameter; s mm	2.14 ± 0.09	2.10 ± 0.10	3.81 ± 0.09^†††^	3.28 ± 0.08^†††,^∗∗∗
Fractional Shortening %	41.7 ± 1.6	41.5 ± 2.1	15.3 ± 1.0^†††^	22.7 ± 1.3^†††,^∗∗∗
Relative Wall Thickness	0.55 ± 0.03	0.54 ± 0.02	0.38 ± 0.01^†††^	0.49 ± 0.02^†,^∗∗∗

**B-mode - Left Ventricle**				

Endocardial Area; d mm^2^	11.9 ± 0.4	11.0 ± 0.4	17.7 ± 0.6^††††^	15.3 ± 0.3^††††,^∗∗∗∗
Endocardial Area; s mm^2^	5.6 ± 0.3	4.9 ± 0.3	13.5 ± 0.6^††††^	10.8 ± 0.3^††††,^∗∗∗∗
Epicardial Area; d mm^2^	26.2 ± 0.7	25.2 ± 0.5	31.9 ± 0.8^††††^	30.9 ± 0.5^††††^
Epicardial Area; s mm^2^	21.3 ± 0.7	21.0 ± 0.6	28.5 ± 0.8^††††^	27.4 ± 0.5^††††^
Wall Thickness; d mm	0.94 ± 0.02	0.96 ± 0.02	0.81 ± 0.02^†††^	0.93 ± 0.01∗∗∗∗
Endocardial Major Axis; d mm	5.86 ± 0.14	5.69 ± 0.11	6.38 ± 0.11^††^	6.18 ± 0.09^††^
Endocardial Major Axis; s mm	4.89 ± 0.13	4.60 ± 0.14	5.90 ± 0.11^††††^	5.51 ± 0.09^††††,^∗∗
Volume; d μL	58.4 ± 2.5	52.5 ± 2.5	94.9 ± 4.0^††††^	79.2 ± 2.1^††††,^∗∗∗
Volume; s μL	22.7 ± 1.6	19.4 ± 1.8	67.0 ± 3.5^††††^	50.1 ± 1.8^††††,^∗∗∗∗
Ejection Fraction %	61.4 ± 1.8	64.4 ± 2.2	30.1 ± 1.6^††††^	36.9 ± 1.3^††††,^∗∗

TM54 mice and non-transgenic littermates (NTG) were treated with 10^11^ vg AAV9sc.PBD or AAV9sc.GFP at 1–3 days of age and analyzed by echocardiography at 16 weeks of age. Relative Wall Thickness = (Anterior Wall; d + Posterior Wall; d)/(Interior Diameter; d). d, diastole; s, systole. Data were analyzed by two-way ANOVA and uncorrected Fisher’s least significant difference post hoc testing for four family comparisons. Data are mean ± SEM ∗ p value for comparison of TM54 cohorts; ^†^ p value for comparison of TM54 vs. NTG for cohorts treated with the same virus. ∗,^†^p ≤ 0.05; ∗∗,^††^p ≤ 0.01; ∗∗∗, ^†††^p ≤ 0.001; ∗∗∗∗, ^††††^p ≤ 0.0001. There were no significant differences among the NTG cohorts.

### AAV9sc.PBD gene therapy improves the cardiac structure of the TM54 DCM mouse

**Figure 3:**
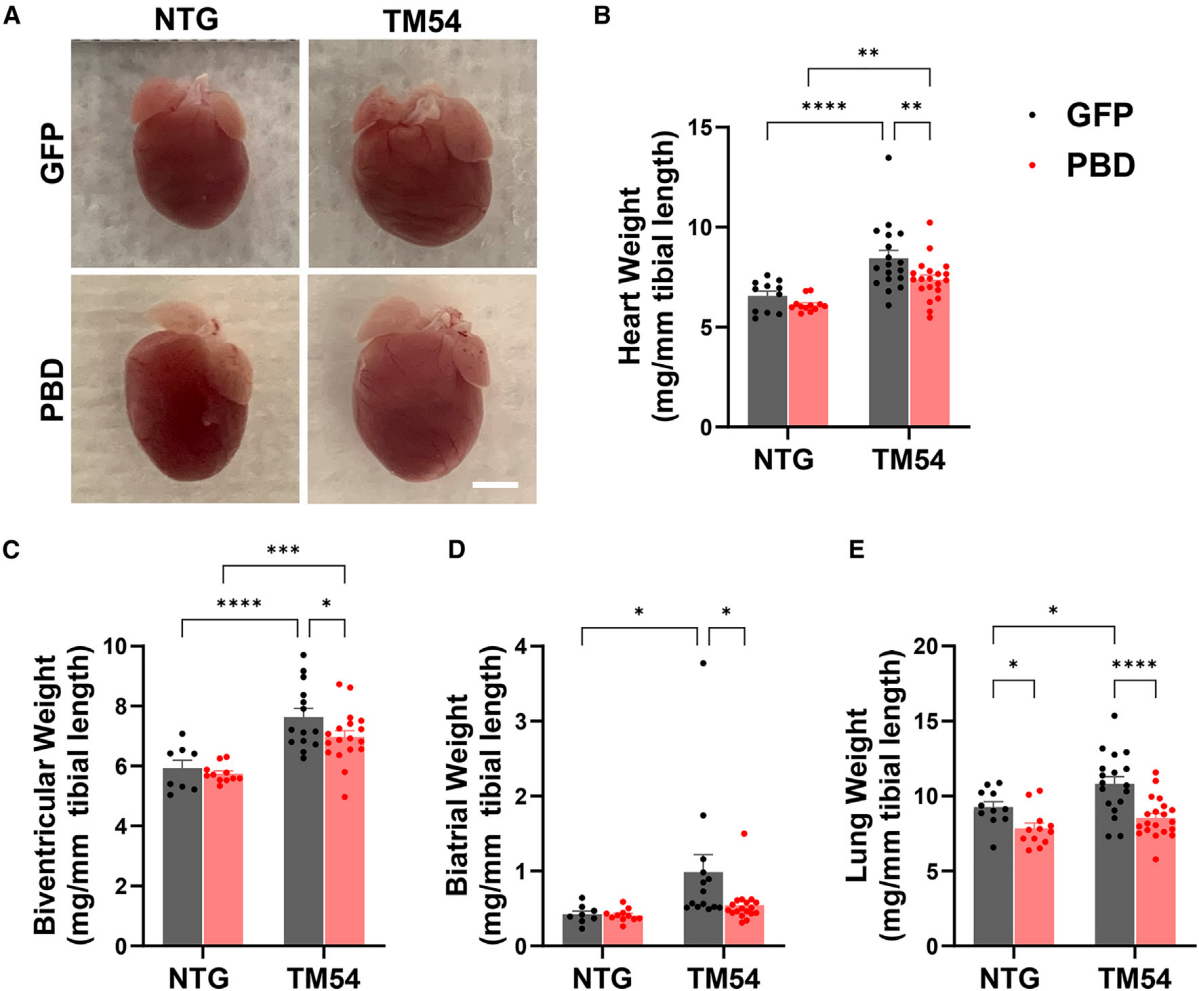
AAV9sc.PBD treatment inhibits cardiac hypertrophy in 16-week-old TM54 mice (A) Images of hearts at the 16-week endpoint. Bar, 2 mm. (B–E) Whole heart, biventricular, biatrial, and wet lung weights at endpoint normalized to tibial length. ∗p < 0.05; ∗∗p < 0.01; ∗∗∗p < 0.001; ∗∗∗∗p < 0.0001. (B) and (E): n = NTG-GFP – 11, NTG – PBD – 12; TM54 – GFP – 18; TM54-PBD – 20. (C) and (D): n = NTG-GFP – 8, NTG – PBD – 11; TM54 – GFP – 14; TM54-PBD – 18. Bars are mean ± SEM. Data analyzed by two-way ANOVA and uncorrected Fisher’s least significant difference post hoc testing.

DCM involves cardiac remodeling resulting in a more spherical shape of the heart (Figure 3A). Consistent with the echocardiographic findings (Figure 2), AAV9sc.PBD-treated TM54 hearts were more ellipsoid in appearance, more similar to those of normal NTG control mice. As expected for a DCM model, the TM54 mouse exhibited ventricular and atrial cardiac hypertrophy (28%, 29%, and 234% increased whole heart, biventricular, and biatrial indexed weight for TM54 vs. NTG-GFP-treated control cohorts, respectively, Figures 3B–3D). Notably, AAV9sc.PBD treatment attenuated the increase in overall heart, biventricular, and biatrial weight by 57%, 39%, and 78%, respectively, consistent with the beneficial effects of PP2A anchoring disruption on cardiac remodeling.

By the 16 weeks of age endpoint, the TM54 line used in this study exhibited little mortality (6% and 2% for TM54 – AAV9sc.GFP and PBD cohorts, respectively). In addition, the increase in wet lung weight, which would indicate heart failure, was small (17%), albeit statistically significant for the control TM54 cohort (p = 0.01, Figure 3E). Nevertheless, the TM54-associated increase in wet lung weight was prevented by AAV9sc.PBD therapy (p < 0.0001 for TM54-PBD vs. TM54-GFP cohorts).

**Figure 4:**
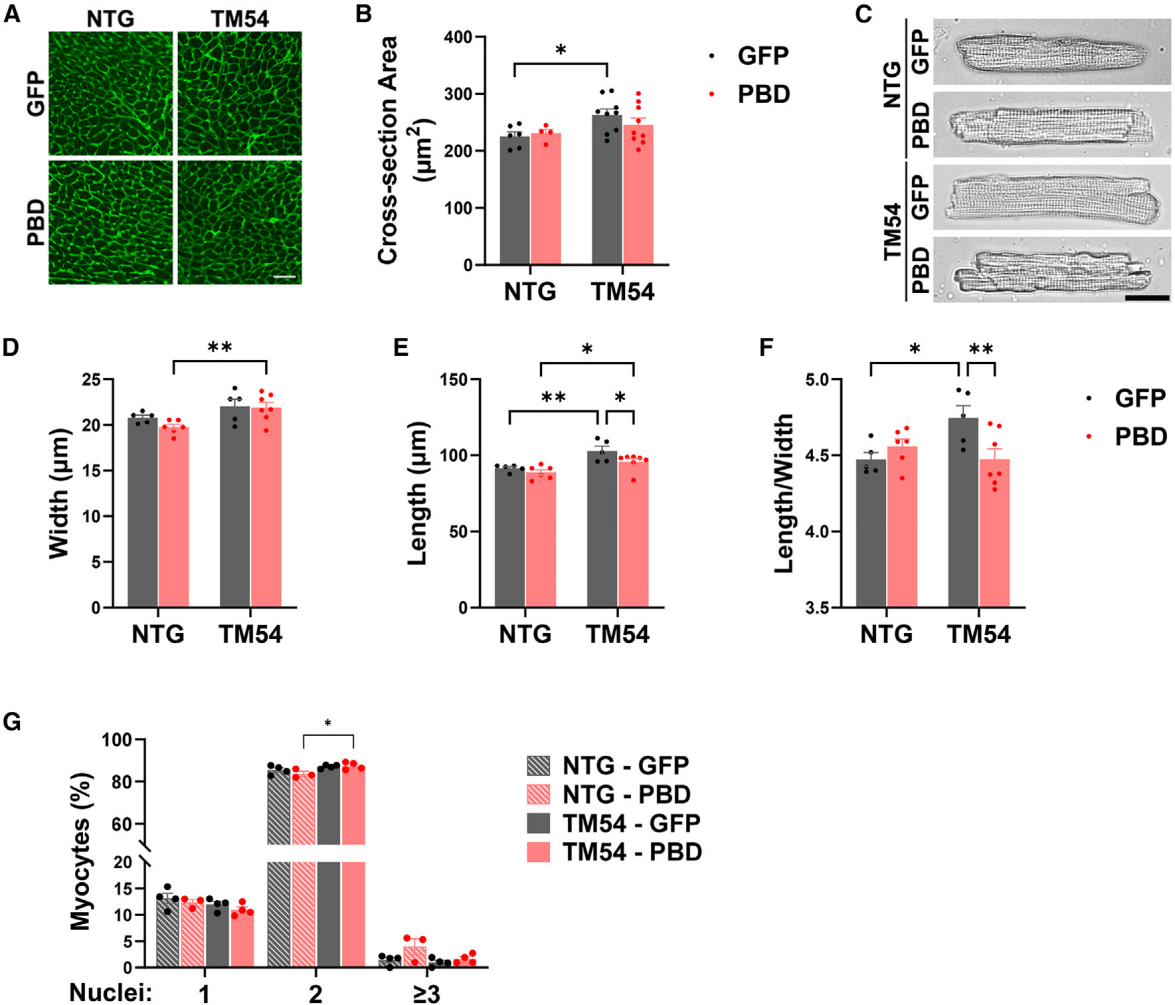
AAV9sc.PBD treatment improves cardiomyocyte morphology in 16-week-old TM54 mice (A) Wheat germ agglutinin-stained left ventricular tissue sections. Bar, 50 μm. (B) Cardiomyocyte cross-section area. n (mice) = NTG-GFP – 6, NTG – PBD – 4; TM54 – GFP – 9; TM54-PBD – 9. (C) Isolated cardiomyocytes. Bar, 25 μm. (D–F) Morphometry of isolated cardiomyocytes. Mean values for individual mice shown; n = NTG-GFP – 5, NTG – PBD – 6; TM54 – GFP – 5; TM54-PBD – 7. (G) Number of nuclei per cardiomyocyte. n = 3–4. ∗p < 0.05; ∗∗p < 0.01. Data analyzed by two-way ANOVA and uncorrected Fisher’s least significant difference (B–F) and by Tukey’s (G) post hoc testing.

Cardiomyocytes are roughly cylindrical in shape, and cardiac hypertrophy in DCM involves a non-mitotic growth greater in length than width of cardiomyocytes.^6^ Analysis of the cross-sectional area of myocytes (perpendicular to myocyte long axis) in histologic sections and morphometric analysis of individual isolated cardiomyocytes confirmed that TM54 control AAV9sc.GFP-transduced mice exhibited myocyte hypertrophy, with the expected increase in myocyte length greater than that in width (12% increase in length [p = 0.002] vs. 6% increase in width [p = 0.14] and 17% increase in cross-section area [p = 0.02, proportional to width squared], respectively, in comparison to NTG AAV9sc.GFP controls, Figures 4A–4E). While the growth in myocyte width was not significantly decreased by AAV9sc.PBD treatment, the TM54-associated increase in myocyte length was decreased (TM54-PBD vs. TM54-GFP cohorts, p = 0.03, Figure 4E), and moreover, the TM54-associated increase in myocyte length:width ratio (6% for TM54-GFP vs. NTG-GFP cohorts, p = 0.01, Figure 4F) was completely blocked by AAV9sc.PBD administration (p = 0.006 for TM54-PBD vs. TM54-GFP cohorts). As a control, the isolated myocytes were examined for the number of nuclei per cell, and no differences in multinucleation were detected between AAV9sc.PBD- and AAV9sc.GFP-treated mice (Figure 4G). Together, these results are consistent with the improved cardiac hypertrophy detected gravimetrically and the improved relative wall thickness detected by echocardiography for TM54 mice treated with AAV9sc.PBD.

**Figure 5:**
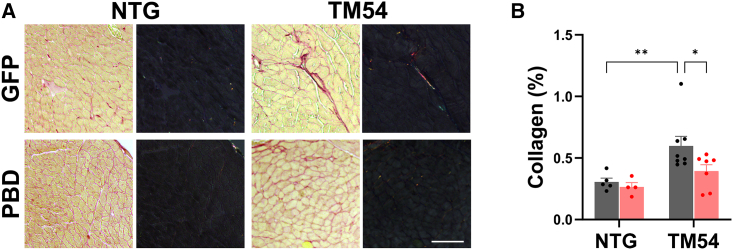
AAV9sc.PBD treatment inhibits myocardial fibrosis in 16-week-old TM54 mice (A) Brightfield (left images) and circularly polarized (right images) light microscopy of picrosirius red-stained left ventricular tissue sections. Bar, 75 μm. (B) Mean collagen content for individual mice measured using circularly polarized light images. n = NTG-GFP – 5, NTG – PBD – 4; TM54 – GFP – 8; TM54-PBD – 7. Bars are mean ± SEM. ∗p < 0.05; ∗∗p < 0.01. Data analyzed by two-way ANOVA and uncorrected Fisher’s least significant difference test.

Pathological cardiac remodeling in DCM also includes progressive myocardial interstitial fibrosis. Interstitial myocardial collagen content detected by picrosirius red staining at endpoint was increased 2-fold in the TM54 AAV9sc.GFP cohort compared with NTG littermates (Figure 5). Reactive fibrosis was decreased 70% by AAV9sc.PBD treatment (p = 0.02 for TM54-PBD vs. TM54-GFP cohorts), demonstrating further the beneficial effects of PP2A anchoring disruption.

### Molecular mechanisms contributing to the benefits of AAV9sc.PBD therapy in DCM

**Figure 6:**
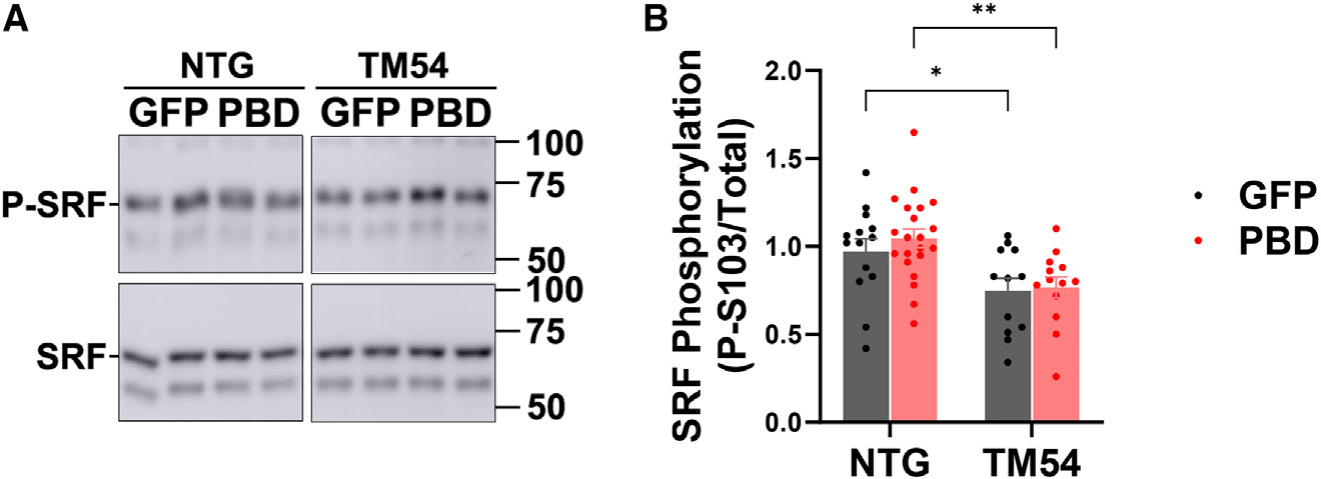
SRF Ser-103 phosphorylation in decreased in 4-week-old TM54 mice (A) Western blot of left ventricular extracts for phospho Ser-103 (top) and total SRF (bottom) protein. (B) Densitometry. n = NTG-GFP – 14, NTG – PBD – 21; TM54 – GFP – 12; TM54-PBD – 13. Bars are mean ± SEM. Data analyzed by two-way ANOVA and uncorrected Fisher’s least significant difference post hoc testing. ∗p < 0.05; ∗∗p < 0.01.

We previously showed that one PP2A substrate at mAKAPβ signalosomes is phosphorylated Ser-103 on the transcription factor serum response factor (SRF).^7^ Notably, SRF Ser-103 phosphorylation comprises an epigenomic switch determining the balance between myocyte growth in width and length, such that increased Ser-103 phosphorylation promotes a decrease in myocyte length:width ratio.^7^ Consistent with a previous report,^13^ we found that SRF Ser-103 phosphorylation was decreased in mice as young as 4 weeks of age (24% less for TM54-GFP vs. NTG-GFP cohorts, p = 0.02, Figure 6), an age at which the DCM phenotype was already apparent (left ventricular ejection fraction for AAV9sc.GFP cohorts [%]: NTG - 58 ± 2, TM54 - 35 ± 5, *n* = 10, 5; p < 0.001 by t test). We did not, however, detect an increased SRF Ser-103 phosphorylation in whole heart extracts prepared from the AAV9sc.PBD TM54 mouse cohorts (see discussion below).

While an increase in SRF phosphorylation was not detected for AAV9sc.PBD-treated TM54 mice, at the 16-week endpoint gene expression for key regulators and markers of cardiac remodeling was significantly improved by the AAV therapy (Table 2). mRNA levels for *Atp2a2,* which encodes sarco(endo)plasmic reticulum calcium-ATPase 2 pump (SERCA2) and is typically down-regulated in heart failure,^14^ were decreased in TM54 AAV9sc.GFP control mice, but restored in AAV9sc.PBD-treated mice. Likewise, the expression of *Pln*, which encodes phospholamban and is often observed to be down-regulated in failing hearts,^15^ was decreased in TM54 AAV9sc.GFP control mice, but restored in AAV9sc.PBD-treated mice. *Myh6* (α-myosin heavy chain) and *Myh7* (β-myosin heavy chain) are differentially regulated in heart failure,^16^ and mRNA levels for these genes were oppositely modulated by the TM54 transgene and AAV9sc.PBD treatment. In addition, *Tgfb2* (transforming growth factor β2), which promotes myocardial fibrosis and hypertrophy, and *Nppa* (atrial natriuretic peptide) and *Nppb* (brain natriuretic peptide), which are markers of cardiac stress,^17^ were increased in expression in TM54 AAV9sc.GFP control mice and significantly reduced toward control NTG levels in AAV9sc.PBD-treated TM54 mice. Together, the normalized expression of these genes supports the conclusion that AAV9sc.PBD therapy is beneficial in TM54 DCM.

**Table 2:** AAV9sc.PBD treatment improves cardiac gene expression in the TM54 DCM mouse

Gene	NTG-GFP	NTG-PBD	TM54-GFP	TM54-PBD
*Acta1*	1.00 ± 0.11	1.00 ± 0.10	3.01 ± 0.38^††††^	2.73 ± 0.39^†††^
*Atp2a2*	1.00 ± 0.03	1.15 ± 0.03∗∗	0.77 ± 0.05^††††^	0.97 ± 0.03^††,^∗∗∗
*Col1a1*	1.00 ± 0.06	0.90 ± 0.05	1.69 ± 0.19^††††^	1.52 ± 0.08^†††^
*Col3a1*	1.00 ± 0.05	0.87 ± 0.07	1.25 ± 0.16	1.28 ± 0.07^††^
*Fos*	1.00 ± 0.32	0.67 ± 0.12	0.78 ± 0.12	0.58 ± 0.08
*Gapdh*	1.00 ± 0.03	1.01 ± 0.05	1.00 ± 0.02	1.02 ± 0.02
*Jun*	1.00 ± 0.07	1.19 ± 0.05∗	0.83 ± 0.06^†^	0.99 ± 0.06^†a^
*Junb*	1.00 ± 0.10	0.83 ± 0.03	0.97 ± 0.05	0.94 ± 0.06
*Myh6*	1.00 ± 0.03	1.13 ± 0.04∗	0.77 ± 0.05^†††^	0.94 ± 0.04^††,^∗∗
*Myh7*	1.00 ± 0.15	0.87 ± 0.07	6.54 ± 1.00^††††^	4.12 ± 0.70^†††,^∗∗
*Nppa*	1.00 ± 0.34	0.32 ± 0.06	6.61 ± 2.51^††^	1.51 ± 0.24∗∗
*Nppb*	1.00 ± 0.17	0.87 ± 0.07	1.61 ± 0.18^††^	1.18 ± 0.10∗
*Pln*	1.00 ± 0.05	1.05 ± 0.03	0.84 ± 0.07^†^	0.99 ± 0.03∗
*Ryr2*	1.00 ± 0.05	1.05 ± 0.05	0.73 ± 0.05^†††^	0.87 ± 0.06^†^
*Srf*	1.00 ± 0.04	0.97 ± 0.04	0.97 ± 0.02	1.00 ± 0.03
*Tgfb2*	1.00 ± 0.08	0.88 ± 0.03	2.47 ± 0.41^††††^	1.45 ± 0.08∗∗
*Trpc6*	1.00 ± 0.06	1.05 ± 0.04	0.86 ± 0.04^†^	0.95 ± 0.05

Mouse left ventricular total RNA from 16-week-old mice was assayed by NanoString technology for mRNA levels for the indicated genes. n = 9 for all cohorts. All data (mean ± SEM) are fold-expression compared with the non-transgenic GFP (NTG-GFP) cohort and are normalized to the expression of *Hprt*. p values were calculated using 2-way ANOVA for individual gene datasets with uncorrected Fisher’s least significant difference post hoc testing for 4-way comparisons. Data are mean ± SEM. ^†^ p value for TM54 vs. NTG for same AAV; ∗ p value for PBD vs. GFP for same mouse genotype. ∗p < 0.05; ∗∗,^††^p < 0.01; ∗∗∗,^†††^p < 0.001; ^††††^p < 0.0001; ^a^ p = 0.054.

## Discussion

The main finding of this study is that the AAV9sc.PBD biologic, which expresses an mAKAPβ-derived PP2A anchoring disruptor peptide, improved the cardiac structure and function of the TM54 DCM mouse. Cardiomyocyte-selective PBD expression improved cardiac systolic function, including left ventricular ejection fraction and end-systolic volume. In addition, AAV9sc.PBD attenuated cardiac remodeling, including improved end-diastolic volume, relative wall thickness, cardiac hypertrophy, myocardial fibrosis, pathological gene expression, and myocyte length:width ratio at the cellular level. Together, these results provide proof-of-concept that AAV9sc.PBD will inhibit the development of the DCM phenotype due to *Tpm1* mutation.

mAKAPβ-bound PP2A can promote pathological eccentric cardiac hypertrophy by at least two different mechanisms. First, signalosome-dependent dephosphorylation of SRF S^103^ alters SRF enhancer binding and gene expression, increasing the length:width ratio of hypertrophic adult rat ventricular myocytes *in vitro*.^7^ Similar to the A-Kinase Interacting Protein 1 (AKIP1) transgenic mouse that also exhibits myocyte elongation,^18^ phosphorylation of SRF S^103^ and the upstream SRF kinase p90 ribosomal S6 kinase (RSK3) has been observed to be reduced in the myocardium of TM54 mice^13^; we confirmed here reduced SRF phosphorylation in TM54 mice. Inappropriate activation of mAKAPβ-bound PP2A resulting in decreased SRF phosphorylation could contribute to the eccentric hypertrophy in these models. We were not, however, able to detect an increase in SRF phosphorylation in TM54 whole heart extracts following AAV9sc.PBD treatment. Although it is possible that AAV9sc.PBD reduced myocyte length:width ratio in TM54 mice independently of increased SRF phosphorylation, this negative result could be due to an incomplete cellular penetrance of AAV transduction. It is possible that while precluding detection of a significant restoration of SRF phosphorylation in whole tissue extracts, AAV9sc.PBD improved SRF phosphorylation and cardiac function following threshold delivery to a subset of ventricular myocytes, similar to the beneficial effects of partial dystrophin restoration in Duchenne’s muscular dystrophy.^19^

A second mechanism by which mAKAPβ-bound PP2A can contribute to pathological cardiac remodeling is the dephosphorylation of mAKAPβ-bound type 4D3 phosphodiesterase (PDE4D3).^11^ Dephosphorylation of PDE4D3 residue S^54^ inhibits the activity of the phosphodiesterase, creating a feedforward loop potentiating local cAMP-dependent PKA signaling. mAKAPβ-bound PKA is required for the induction of myocyte hypertrophy through local crosstalk with other relevant signaling pathways such as the calcineurin-NFAT pathway.^20^^,^^21^ Interestingly, *Pde4d* gene knockout in mice resulted in a spontaneous DCM phenotype, as well as worse outcome after myocardial infarction.^22^ A limitation of this study is that we were unable to assay reliably PDE4D3 phosphorylation in mouse tissue, and accordingly, we could not determine whether the TM54 transgene or AAV9sc.PBD treatment affected PDE4D3 phosphorylation. Besides SRF and PDE4D3, other components of mAKAPβ signalosomes could be targets for mAKAPβ-bound PP2A and PBD anti-remodeling activity, including, for example, adenylyl cyclase 5, which is inhibited by PKA-dependent phosphorylation.^23^^,^^24^ It is also possible that the diffusely localized PBD peptide displaces PP2A containing B56δ holoenzyme (the isoform known to bind mAKAPβ^11^) from other binding partners besides mAKAPβ, thereby inhibiting the dephosphorylation of additional potentially relevant substrates in other intracellular compartments. Future research will be necessary to identify the cardiomyocyte PP2A substrates whose phosphorylation is increased by PBD expression.

While we were unable to identify the PP2A substrates directly affected by AAV9sc.PBD administration, the improvement in the cardiac structure and systolic function of the TM54 mouse correlated with normalized cardiomyocyte morphology, which would decrease ventricular wall stress, as well as by an improvement in relevant gene expression. For example, canonical markers of pathological remodeling such as *Nppa, Nppb, Myh6,* and *Myh7* were normalized in expression by AAV9sc.PBD. *Nppa* and *Nppb*, which express atrial and brain natriuretic peptide, respectively, are compensatory markers for increased wall stress, and their decreased expression would be expected given the improved structure and function of the AAV9sc.PBD-treated TM54 mice.^25^ In addition, the attenuation of *Tgfb2* expression presumably contributed to the decreased myocardial hypertrophy and fibrosis in AAV9sc.PBD-treated TM54 mice.^17^ Notably, the improved cardiac contractile function of the TM54 AAV9sc.PBD cohort can be attributed at least in part to restoration of *Atp2a2* (SERCA2) expression. SERCA2 is responsible for removal of cytosolic Ca^2+^ during diastole and is down-regulated in heart failure.^14^ Restored SERCA2 expression, which improves sarcoplasmic reticulum loading and excitation-contraction coupling, has been proposed as an approach to improve the systolic dysfunction in HFrEF, and gene therapy trials are now under way to test the improvement provided by directly increasing expression of SERCA2a in heart failure (ClinicalTrials.gov ID NCT04703842 and NCT06061549).

We suggest that the results obtained for the TM54 mouse may reflect the potential of PP2A anchoring disruptor therapy to treat DCM of different etiologies, especially given the beneficial effects AAV9sc.PBD, as well as mAKAPβ scaffold gene targeting, exhibited previously in ischemic cardiomyopathy.^7^^,^^9^ The isoform of PP2A regulatory B-subunit found to be a constituent of mAKAPβ-bound PP2A holoenzyme is B56δ (PPP2R5D), an isoform associated with DCM.^11^ DeGrande et al. showed that B56δ mRNA levels were increased >6-fold in human non-ischemic heart failure.^26^ In addition, they found that in canines B56δ protein was increased in tachy-pacing-induced non-ischemic heart failure. PP2A B56δ holoenzyme is activated by PKA phosphorylation, as potentiated by association with mAKAPβ perinuclear signalosomes.^11^ Ranieri et al. showed that β-adrenergic signaling *in vitro* and pressure overload in mice increased the level of S^573^-phosphorylated B56δ, a mechanism that would be consistent with neurohormonal activation in HFrEF and the beneficial effects of β-blockade in ischemic and non-ischemic DCM.^27^ Together, these findings suggest that targeting mAKAPβ-bound PP2A action will be beneficial across diverse, if not all DCM mutations. Moreover, we note that the endpoint of this study was when mice were 16 weeks of age, before heart failure was prominent in the control AAV9sc.GFP-treated TM54 cohort. As the difference in echocardiographic parameters between control and AAVsc.PBD-treated TM54 mice increased between 12 and 16 weeks of age (Figure 1B), we suspect that the observed improvement in cardiac function provided by AAV9sc.PBD underestimates the potential beneficial effect of the gene therapy in DCM, as might be observed in later-stage disease. Taken together with evidence of PP2A upregulation in DCM, these results provide impetus for the establishment of an AAV9sc.PBD translational pipeline for the treatment of this disease, including the testing of AAV9sc.PBD in additional models of DCM based upon different genetic mutations.

## Materials and methods

### Animal subjects

Animal research was approved by the Administrative Panel on Laboratory Animal Care Institutional Animal Care and Use Committee at Stanford University. FVB/N-Tg(Myh6-Tpm1∗E54K) “TM54” mice were kindly provided by Dr. Beata Wolska and Dr. David Wieczorek and are now available as strain #035610 at the Jackson Laboratory (Bar Harbor, ME).^12^ Mice were genotyped by polymerase chain reaction using the following primers: Myh6 Forward: 5′-GCC CAC ACC AGA AAT GAC AGA-3′ and Tpm1 Reverse: 5′-TCC AGT TCA TCT TCA GTG CCC-3′ (236-base pair [bp] product); GAPDH internal control sense: 5′-AGC GAG CTC AGG ACA TTC TGG-3′ and antisense: 5′-CTC CTA ACC ACG CTC CTA GCA-3′ (494 bp). Both male and female mice were included in all cohorts. Data for pooled sex cohorts are shown as there was no evidence of significant sexual dimorphism in this study. Masking of cohorts was provided by assigning a random number to each mouse by ear tag, such that identification of mouse cohort was not revealed until *in vivo* and postmortem analyses were complete. A formal power analysis was not performed for the studies in this project. Mice were not selected for AAV delivery or expression before analysis.

### Adeno-associated virus

Self-complementary serotype 9 AAV that expresses under the control of the chicken cardiac troponin T promoter myc-tagged rat mAKAP aa 2134–2314 PBD polypeptide or myc- and His_6_-tagged GFP control, AAV9sc.PBD and AAV9sc.GFP, respectively, were as previously described and produced by the University of Pennsylvania Vector Core.^7^ AAV9sc was injected intraperitoneally (1 × 10^11^ vg i.p.) into 2- to 3-day-old TM54 and wild-type littermate mice.

### Echocardiography

Mice, minimally anesthetized with 1%–3% isoflurane, were studied by transthoracic echocardiography using a Vevo 3100 High-Resolution Imaging System (VisualSonics, Toronto, ON, Canada). For M-mode echocardiography, calculated parameters from at least three cardiac cycles were as follows: FS, fractional shortening = (LVID; d – LVID; s)/(LVID; d) and relative wall thickness = (LVAW; d + LWPW; d)/(LVID; d) in which LVID, LVAW, and LVPW are left ventricular interior diameter, anterior wall thickness, and posterior wall thickness, respectively, and d and s refer to diastole and systole, respectively. For B-mode echocardiography using both short and long axis parasternal imaging: LV Volume = 5/6∗(Endocardial Area)∗(Endocardial Major Axis); LV Wall Thickness = average wall thickness as calculated from the epicardial and endocardial area tracings; and ejection fraction = (Volume; d – Volume; s)/Volume; d. All parameters were measured or calculated using VevoLAB software (VisualSonics).

### Histochemistry

Heart tissue was fixed in 3.7% formaldehyde. De-paraffinized 5- or 8-μm tissue sections were stained using the picrosirius red Stain Kit (Polysciences, Warrington, PA) and Alexa Fluor 555 Wheat Germ Agglutinin conjugate (Invitrogen, Waltham, MA) as recommended by the manufacturers, as previously described.^7^ The cross-section area of >150 myocytes in >3 distinct regions of the left ventricle were measured per heart using the wheat germ agglutinin sections following imaging at 200x using a Leica DM4000 fluorescent microscope and a DFC3000G camera. Collagen content in myocardium, excluding the area around blood vessels, was assayed using the picrosirius red-stained sections by polarized light microscopy at ×200 magnification and imaging of the entire left ventricle using the Leica DM4000 microscope and a DMC2900 color camera. WGA transverse sections for cross-section area and collagen content were measured using NIH ImageJ or Leica LAS software.

### Adult mouse myocyte isolation by Langendorff perfusion

Adult myocytes were isolated from mouse hearts and fixed in suspension in perfusion buffer containing 3.7% formaldehyde, before morphometric analysis by light microscopy, as previously described.^7^

### Western blotting

Protein lysates from whole heart tissue were analyzed by western blot using antibodies for myc tag (custom rabbit antibody FL102),^7^ SRF (Rabbit, #5147, Cell Signaling, Danvers, MA), and phospho-SRF (Rabbit, #4261, Cell Signaling, Danvers, MA) as previously described.^7^

### NanoString assays

The NanoString assay is based on direct, multiplexed measurement of gene expression without amplification, utilizing fluorescent molecular barcodes and single molecule imaging to quantify multiple transcripts in a single reaction. Total heart RNA was hybridized in solution to a custom target-specific codeset (NanoString Technologies, Seattle, WA), and mRNA species counted as previously described.^7^ Datasets for each RNA sample were background-subtracted and normalized by *Hprt* mRNA levels using nSolver 4 software. Probe sequences are available upon request.

### Statistical analysis

The “n” refers to the number of individual mice. All data are expressed as mean ± SEM. Statistics were computed using Prism 10 (Graphpad, San Diego, CA). For experiments involving 2 × 2 design, significance was determined for comparison of cell means with others in its row or column by two-way ANOVA and uncorrected Fisher’s least significant difference test. For NanoString gene expression data, the expression of individual genes was similarly compared across the four mouse cohorts without adjustment for the number of genes assayed, as the genes were not selected at random, and the effects were not expected to be independent. For experimental designs involving >2 time points or conditions, significance was determined by two-way ANOVA and Tukey’s multiple comparison test.

## Data and code availability

The datasets supporting the conclusions of this article are included within the article.
